# A gene expression atlas for kiwifruit (*Actinidia chinensis*) and network analysis of transcription factors

**DOI:** 10.1186/s12870-021-02894-x

**Published:** 2021-02-27

**Authors:** Lara Brian, Ben Warren, Peter McAtee, Jessica Rodrigues, Niels Nieuwenhuizen, Asher Pasha, Karine M. David, Annette Richardson, Nicholas J. Provart, Andrew C. Allan, Erika Varkonyi-Gasic, Robert J. Schaffer

**Affiliations:** 1 The New Zealand Institute for Plant and Food Research Ltd (Plant & Food Research), Private Bag 92169, Auckland, 1146 New Zealand; 2 School of Biological Science, The University of Auckland, Private Bag 92019, Auckland, 1146 New Zealand; 3 Department of Cell & Systems Biology / Centre for the Analysis of Genome Evolution and Function, University of Toronto, 25 Willcocks St, Toronto, ON M5S 3B2 Canada; 4 The New Zealand Institute for Plant and Food Research Ltd (Plant & Food Research), 121 Keri Downs Road, Kerikeri, 0294 New Zealand; 5 The New Zealand Institute for Plant and Food Research Ltd (Plant & Food Research), 55 Old Mill Road, Motueka, 7198 New Zealand

**Keywords:** Actinidia, eFP browser, Transcription factors

## Abstract

**Background:**

Transcriptomic studies combined with a well annotated genome have laid the foundations for new understanding of molecular processes. Tools which visualise gene expression patterns have further added to these resources. The manual annotation of the *Actinidia chinensis* (kiwifruit) genome has resulted in a high quality set of 33,044 genes. Here we investigate gene expression patterns in diverse tissues, visualised in an Electronic Fluorescent Pictograph (eFP) browser, to study the relationship of transcription factor (TF) expression using network analysis.

**Results:**

Sixty-one samples covering diverse tissues at different developmental time points were selected for RNA-seq analysis and an eFP browser was generated to visualise this dataset. 2839 TFs representing 57 different classes were identified and named. Network analysis of the TF expression patterns separated TFs into 14 different modules. Two modules consisting of 237 TFs were correlated with floral bud and flower development, a further two modules containing 160 TFs were associated with fruit development and maturation. A single module of 480 TFs was associated with ethylene-induced fruit ripening. Three “hub” genes correlated with flower and fruit development consisted of a HAF-like gene central to gynoecium development, an ERF and a DOF gene. Maturing and ripening hub genes included a KNOX gene that was associated with seed maturation, and a GRAS-like TF.

**Conclusions:**

This study provides an insight into the complexity of the transcriptional control of flower and fruit development, as well as providing a new resource to the plant community. The Actinidia eFP browser is provided in an accessible format that allows researchers to download and work internally.

**Supplementary Information:**

The online version contains supplementary material available at 10.1186/s12870-021-02894-x.

## Background

Global transcriptomic approaches are a common tool used to obtain a better understanding of gene function and regulation. The composition of the transcriptome is the result of a dynamic balance between chromatin state, the activation of gene expression by transcription factors (TFs) and the speed of transcript degradation. The combination of good genomic information and robust gene models paves the way for systematic and consistent gene and gene family naming. This combined with other genomics tools, such as Electronic Fluorescent Pictograph (eFP) browsers [1] to help visualise where a gene is expressed, allows faster identification of gene function in different species. To date, eFP browsers have been successfully developed in plants such as Arabidopsis [1], tomato [2], strawberry [3], and pineapple [4].

TFs are one of the largest groups of genes in a genome; in Arabidopsis there are over 1500 TFs described, belonging to a number of different classes representing 5% of all genes [5]. In other species TFs represent 3–5% of coding genes, with function often conserved across species [6]. TFs have been grouped into 57 different classes [5] with some classes having multiple types of DNA binding domains. Each class of TF is represented by a gene family. These gene families vary in size from species to species depending on events such as individual gene and genome duplications, leading to expansions of certain or most families [6]. In higher plants the MYB, bHLH and Zinc finger classes of TF contain many hundreds of members [6]. There are numerous examples demonstrating the strong evolutionary maintenance of TF primary protein structure across species, with the homologous genes having a similar gene function. This allows researchers to predict function by homology [7].

The MADS-box containing TFs form arguably one of the best understood classes of TF. Members of the MADS-box gene family, including the well-known floral organ structure ABCE TFs, determine many aspects of plant development [8, 9]. Even though the fruiting bodies of Angiosperms are homoplasious, with fleshy fruit evolving numerous times within many plant families the function of these genes appear conserved [10]. Angiosperm flower structure and fruiting bodies are remarkably conserved, with whorls of sepals, petals, stamens and carpels [8]. The MADS protein sequence is also conserved with many examples within plants demonstrating similar control mechanisms across many species [7, 11].

Kiwifruit are part of the *Actinidiaceae* which is a basal family within Ericales [12], and contains the genus *Actinidia* comprising of a number of economically important fruit species such as *Actinidia chinensis* var. *deliciosa* (green kiwifruit), *A. chinensis* var. *chinensis* (gold and red kiwifruit) and *A. arguta* (hardy kiwifruit or kiwiberries). The green ‘Hayward’ kiwifruit is hexaploid, while a commercially released yellow fleshed variety *A. chinensis* var. *chinensis*, ‘Hort16A’, and the red fleshed *A. chinensis* var. *chinensis* ‘Hongyang’ are large fruiting diploid genotypes making them ideal for understanding molecular processes in *Actinidiaceae*. More recently a new *Pseudomonas syringae* pv*. actinidiae* (*Psa*) tolerant tetraploid gold variety, ‘Zesy002’, has replaced ‘Hort16A’ in the markets. The two diploid cultivars have been used to understand the molecular control of many aspects of development including flowering, fruit ripening, colour and flavour development [13–16]. Genomics tools such as CRISPR gene editing have been successfully used to edit the floral repressors in ‘Hort16A’ to create a small fruiting plant that can be used to rapidly test gene function in fruit, further building on their utility [17].

The first draft kiwifruit genome was of *A. chinensis* ‘Hongyang’, published in 2013 [18], paving the way for genomics in *Actinidiaceae*. More recently a second *A. chinensis* genome of a more inbred related genotype, Red5, further improved the construction and importantly manual annotation of gene models [19]. The manual annotation of the kiwifruit genome improved the quality of the published computer predicted gene models, and provided a quality resource for future gene mining. Here we build on these data by identifying TF genes, analysing their expression over a number of tissues and providing an eFP Browser tool to analyse gene expression.

## Results

### Mining of transcription factor gene families

**Fig. 1:**
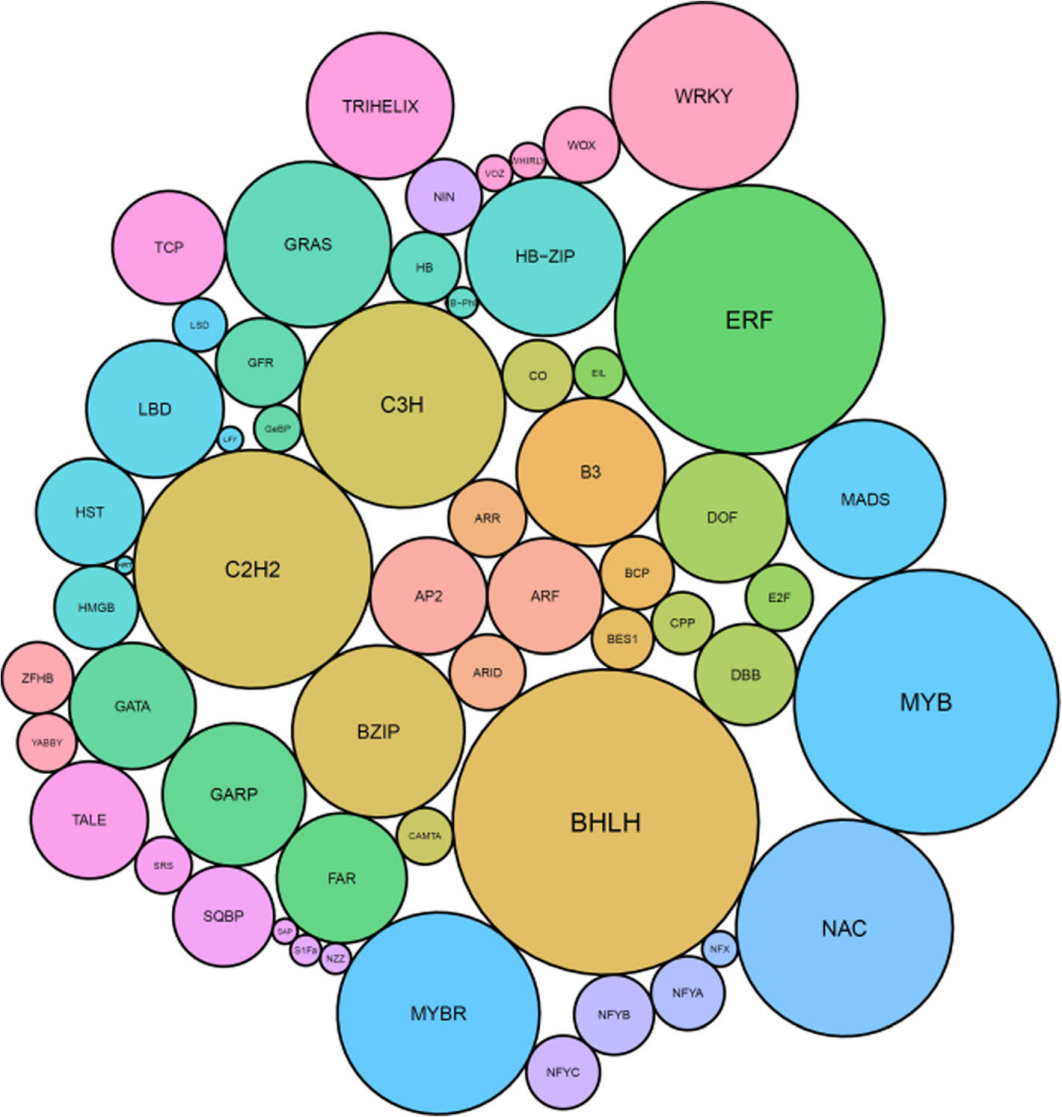
Composition of the different classes of the 2839 transcription factors. Size of circles represent the number of genes within each class

Using the kiwifruit manually annotated gene models [19], protein translations containing InterPro DNA binding domains (http://www.ebi.ac.uk/interpro) were identified. These were manually checked, resulting in 2839 gene models with at least one TF domain in 61 TF classes in 32 global classes (Fig. 1, Table 1, Additional data 1). The most abundant global class of TFs in kiwifruit was the Zinc finger class, represented by 571 genes in 11 different gene classes, followed by 428 genes with MYB domains within four different classes of genes, and 333 bHLH genes within two different classes of genes (Table 1, Additional data 1). In total the 2839 TFs represented 8.6% of the annotated genes in the kiwifruit genome.

**Table 1:** Summary of transcription factors

Major classes	total	class		class		class		class	
Zn finger domain	**571**	C2H2	177	CO	16	DOF	53	C3H	130
		SQBP	32	GATA	50	VOZ	4	DBB	32
		LSD	9	NF	58	SRS	10		
MYB domain	**428**	MYBR	126	MYB	219	ARR	19	GARP	64
BHLH domain	**333**	BHLH	293	TCP	40				
AP2 domain	**270**	ERF	227	AP2	43				
HB domain	**176**	HD-ZIP	79	WOX	18	HD-PHD	3	TALE	44
		HB	16	ZFHD	16				
B3 domain	**111**	ARF	42	RAV	12	LAV	57		
MADS domain	**79**	TYPE 1	23	MICK	56				
HMG type	**50**	HMGB	22	ARID	17	YABBY	11		
**Other groups**									
NAC	147	WRKY	116	Trihelix	67	FAR	53	BZIP	100
GRAS	86	BCP	17	BES	12	LFY	2	EIL	8
CATMA	10	E2F	14	CPP	12	GFR	25	GeBP	7
HST	36	LBD	59	NZZ	3	NIN	18	S1Fa	8
SAP	12	HRT	1	WHIRLY	4	PLATZ	17	STAT	0

**Fig. 2:**
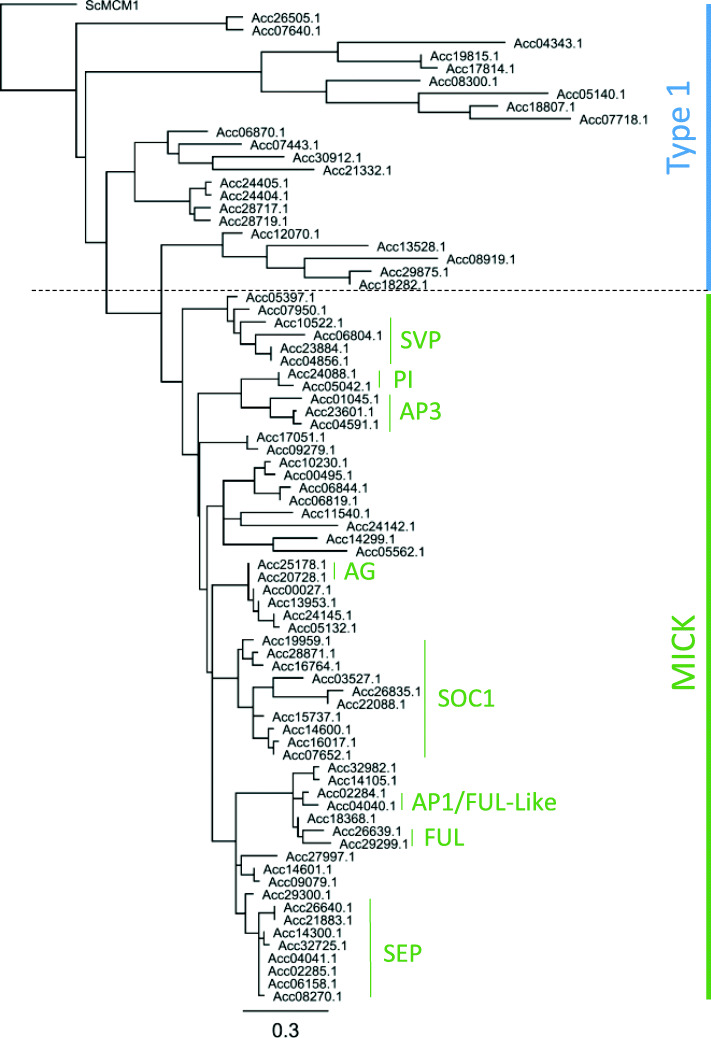
Phylogenetic alignment of predicted proteins containing a MADS DNA binding domain. The two classes of *MADS* genes are shown (Type 1 – Blue bar and MICK – green bar). Names in green type are published genes and groups. A genome duplication has resulted in at least two homeologous gene models for each of the genes

Using the DNA binding domains, each TF class was aligned in a phylogenetic tree and named using the following criteria. Firstly, if the gene had been previously published in the literature, this name was given. For genes not previously published, a sequential naming down an initial phylogenetic tree was used. This naming method allows genes within subclades to have numbers which are close to each other. An exception was made for the ARF genes where the whole family has been well characterised in a number of species [20–22], so a naming convention related to the closest Arabidopsis and tomato homologues [22] was taken. An example cluster of the MADS TF Type 1 and MICK cluster is shown in Fig. 2 and full phylogenies can be found in additional data 2. Within all the gene families, there were typically two closely related genes observed at each branch consistent with the reported genome duplication [19].

In kiwifruit, four classes of TFs have been previously reported, two (the R2R3 class MYBs [23] and WRKYs [24]) were based on a previous version of the kiwifruit genome, the third (the MICK type MADS-box genes) was published using EST sequence data [25] and the fourth (AP2/ERF class of gene) was based on the manually annotated genome [26]. Two other gene families, the R2R3 MYB class and NAC class of TFs are the subject of separate studies and are reported in more depth (Rodrigues et al. submitted, Nieuwenhuizen et al. submitted).

The 96 published WRKY TFs were previously named based on sequential chromosomal locations. Of these, five did not have an Acc annotated gene model and two models (*WRKY95* and *WRKY96*) appear to be splice variants. The five were annotated using the Web Apollo software and Acc numbers assigned. This study identified an additional 21 *WRKY* genes and these new genes were sequentially numbered, bringing the total to 116 *WRKY* genes.

A comprehensive analysis of EST sequences and full length sequence of nine MADS-box genes was reported by Varkonyi-Gasic et al. [25]. Since this study, four *SVP* like genes [27] and eight *SOC1* like genes [28] have been reported. Further mining identified 58 further predicted gene models containing a MADS-box DNA binding domain. The MADS genes separated into two major clades; the Type 1 and MICK type. Previously the MICK type MADS-box genes have been shown to be key regulators of plant development, especially in floral and fruit development. Phylogenetic alignment identified sequences with high similarity to the well-characterised MICK-MADS genes and identified possible homeologous pairs of: *AGAMOUS* (*AG*) like genes, *Acc25178.1* (*MADS28*) and *Acc20728.1* (*MADS29*); *PISTILLATA* (*PI*) like genes, *Acc24088.1* (*MADS11*) and *Acc05042.1* (*MADS12*); and *APETALA1* (*AP1*) like genes, *Acc04040.1* (*MADS40*) and *Acc02284.1* (*MADS41*) (Fig. 2).

### Expression analysis

**Fig. 3:**
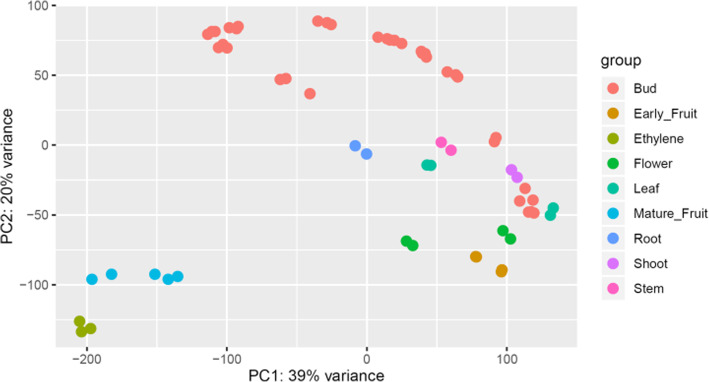
The first two principal components from the top 5000 most differentially expressed genes in a global transcriptomics study of different tissues. The different tissues are depicted by different colours

To establish where and when each of the TFs were expressed, a transcriptomic approach was taken. Global gene expression of different tissues and different plant developmental stages of two cultivars of *A. chinensis* var. *chinensis*, the gold fruited ‘Hort16A’ and ‘Zesy002’ were measured. Sixty-one sets of RNA-seq from root, stem, shoot, leaves, flowers, and early fruit development were combined with RNA-seq reads from fruit development [15] and postharvest [15] were used and a bud development series (Voogd et al. in preparation) (Additional data 3,4). A Principal component analysis (PCA) showed mature fruit and ethylene treated fruit were separated from the other tissues (Fig. 3). Normalised expression patterns of the TFs were extracted. Based on transcripts per million (TPM) values, the majority of TFs were found to have at least one RNA-seq read in one of the datasets, with only 47 (1.67%) having no reads in a single tissue.

### Development of an eFP browser for kiwifruit

**Fig. 4:**
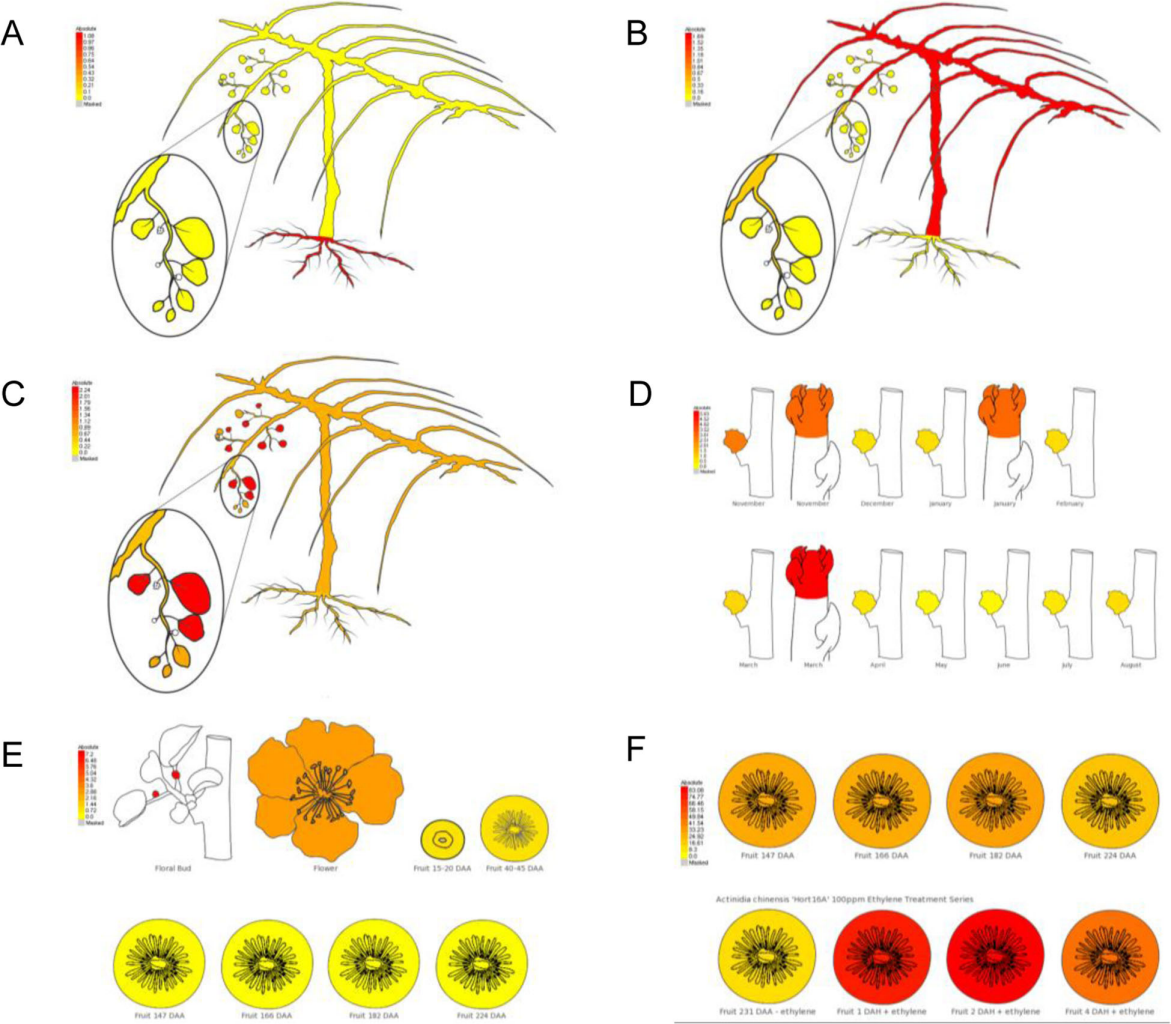
Electronic Fluorescent Pictograph (eFP) browser images of *MADS* related genes showing (**a**) *MADS19* (*Acc00495.1*) root predominant expression, (**b**) *MADS45* (*Acc27997.1*) stem predominant expression, (**c**) *MADS77* (*Acc08919.1*) leaf expression, (**d**) *MADS6* (*Acc10522.1*) bud terminal late bud expression, (**e**) *AG* (*MADS29* - *Acc20728.1*) flower specific expression, (**f**) postharvest ethylene treatment with high expression of a *SEP4/RIN* like gene (*MADS52* – *Acc26640.1*)

To facilitate the visualisation of expression patterns of each gene, an eFP browser, for all gene models, was developed covering different tissues and stages of development. Within the MADS genes, root predominant expression was seen in *MADS12* (*Acc10230.1*) and *MADS19* (*Acc00495.1*) (Fig. 4a.). Consistent with functional analysis in model species, *MADS19* showed closest homology to *AtAGL21* (*At4g37940.1*) which regulates lateral root development in Arabidopsis. A second MADS gene, *MADS45* (*Acc27997.1*) showed predominantly stem and cane expression (Fig. 4b) and showed highest homology to *AGL6*, a floral promoter that negatively regulates the FLC/MAF clade genes and positively regulates FT in Arabidopsis [29]. *MADS77* (*Acc08919.1*) showed leaf specific expression (Fig. 4c). *SVP1* (*MADS6* - *Acc10522.1*) was well expressed in buds (Fig. 4d) and had high homology to the *SVP* Arabidopsis gene (*At2g22540*). Flower predominant expression of *AGAMOUS* like gene *MADS29* (*Acc20728.1*) (Fig. 4e) was observed, while the *RIN/SEP4* [15] gene *MADS52* (*Acc26640.1*) (Fig. 4f) showed postharvest expression.

### Network analysis

**Fig. 5:**
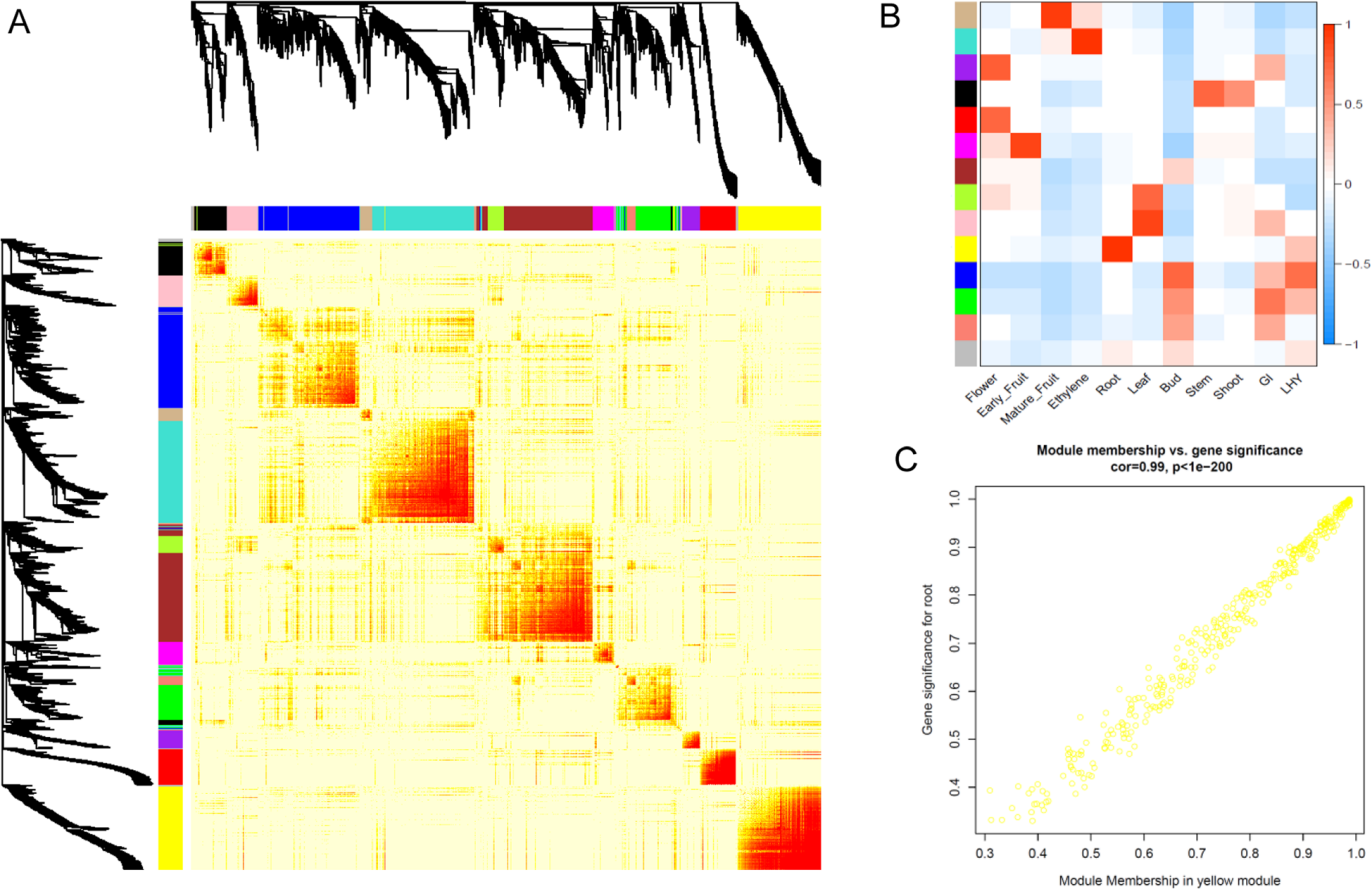
**a** Gene network modules of the 2747 transcription factors. **b** Correlation of the modules with nine different tissues and two selected circadian related genes (afternoon gene *GI* (*Acc12229.1*) and morning gene *LHY* (*MYBR92* Acc24169.1)). **c**. Detailed correlation between the yellow module and the 323 root associated transcription factors

Weighted gene co-expression network analysis (WGCNA) [30] assigned the 2773 expressed TFs to 14 different module colour groups (Fig. 5a). When these were compared to the different tissue types, there were some clear correlations between some modules and different tissue (Fig. 5b). Given that some of the tissue may have been harvested at different times of the day, two circadian genes (a morning MYB related gene, *LHY* [31] – *MYBR92 Acc24169.1*, and an afternoon gene, *GIGANTEA* [32] *Acc12229.1*) were also correlated with the group. The expression pattern of these two genes varied between TPM 2.7 and 28.1 (*MYBR92*) and TPM 2.8 and 46.7 (*GIGANTEA*) suggesting there was some variation in the harvest time (Additional data 5). However this did not seem to affect the network analysis as only weak correlations between these genes and the blue and green modules were observed (Fig. 5b). One of the strongest correlations was root tissues and the yellow module containing 323 TFs (Table 2, Additional data 6) showing 96% correlation (Fig. 5b,c). As expected the root predominant MADS TF (*Acc00495.1 MADS19*) was found within this yellow module.

**Table 2:** Numbers of transcription factors (TF) in each colour network module

Network Module	TF numbers
black	155
blue	451
brown	422
green	203
green yellow	78
grey	40
magenta	100
pink	135
purple	78
red	159
salmon	42
tan	60
turquoise	480
yellow	370

**Fig. 6:**
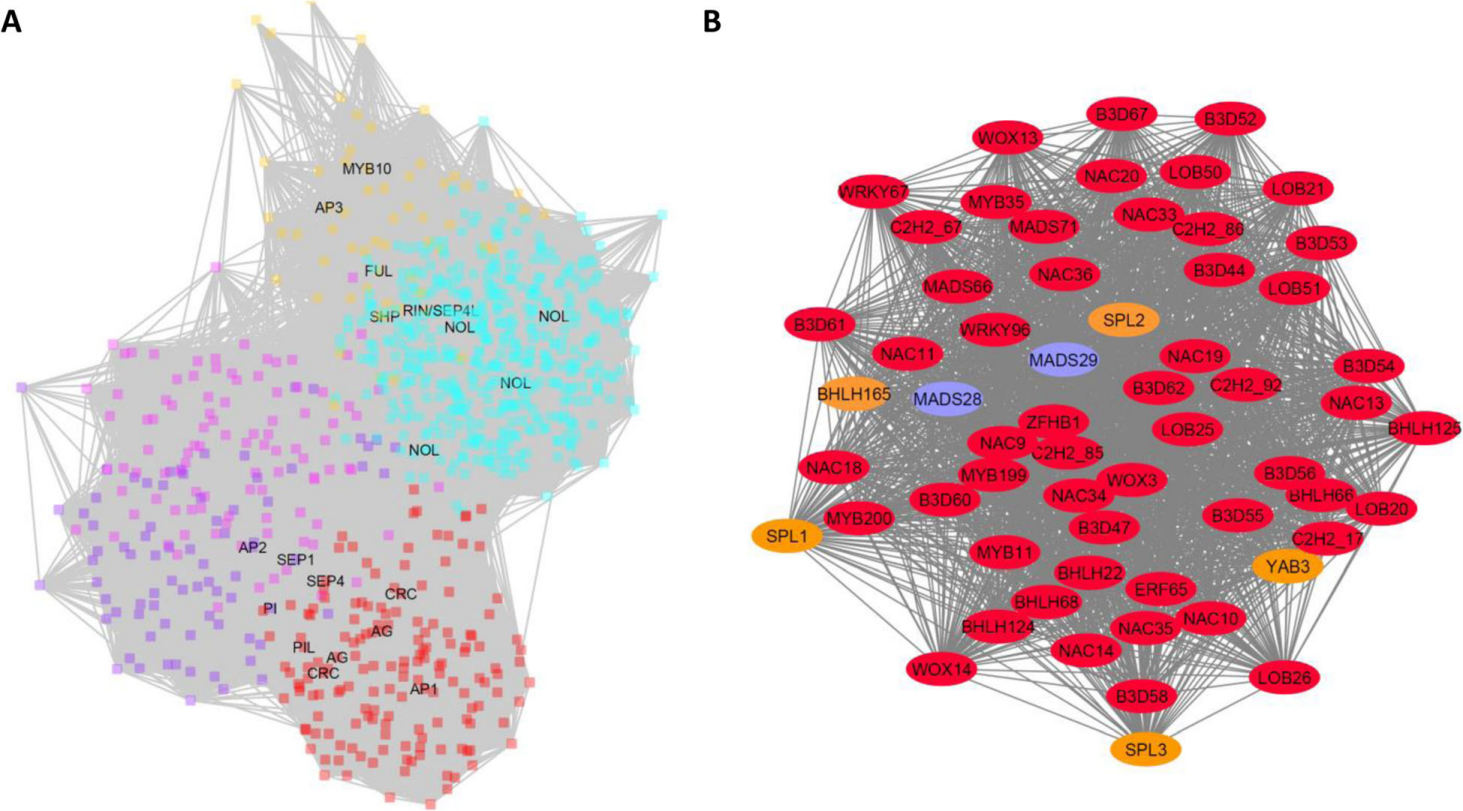
Network maps of flower and fruit development. **a** The red, purple, magenta, tan and turquoise modules with selected transcription factors named, including the *AGAMOUS-like* (*AG*) genes *MADS28*, *MADS29* along with potential homologues of other flowering and fruit development and ripening genes. **b** An adjusted network map of a subset of the red module genes that interact with a weight of > 0.5 with the *AG-like* genes (Blue). Genes that have been shown to be direct targets of *AG* in Arabidopsis are shown in orange and include *CRC* (*YAB3*), *SPOROCYTELESS* (*SPL*), and *HEC1* (*bHLH165*)

Some tissue types showed significant association with more than one network group. Floral buds and open flower showed correlation between the red and purple modules with 159 and 78 genes respectively. A magenta module containing 100 genes had linkages with early fruit development while later fruit development and maturation were more associated with the tan module containing 60 genes. In the turquoise module, 457 TFs were associated with a postharvest ethylene treatment of ripe fruit. A network graph with key known TFs generated for all the flower and fruiting genes (Fig. 6) demonstrated a strong interrelatedness of this selection of genes. Calculated hub genes for the red, purple, magenta, tan and turquoise clusters were *Acc17850.1 - ERF65, Acc28494.1 - bHLH281, Acc18135.1 - DOF43, Acc15461.1 - KNOX3, Acc20237.1 - GRAS13* respectively. The most similar *Arabidopsis* gene to *bHLH281* is *AT1G25330* - *HALF FILLED* (*HAF*) that specifies reproductive tract development in Arabidopsis [33]. The tan hub gene *KNOX3* is similar to *AtKNAT7* which has been proposed to work with *AtPAP1* (the homologue of which, *Acc00493.1* - *MYB10/75*, is also found in the tan cluster) to develop the seed coat [34].

## Discussion

By combining gene mining and expression analysis of the TF families from kiwifruit we have constructed a gene network for different tissues at different developmental stages during the plant life cycle. Through a close examination of the flower and fruit networks which were associated with the red, purple, magenta, tan and turquoise modules (Fig. 6a), a number of MADS-box TFs with close homology to those characterised in other species were found. In the red module there were 10 MICK MADS-box genes including the previously published *AGAMOUS* (*AG*) gene *MADS29* (*Acc20728.1*) and its homeologue *MADS28* (*Acc25178.1*), a *PISTILLATA*-like gene, *MADS11* (*Acc24088.1*), and two *SEP*-like genes, while the purple module contained five MICK MADS genes including a second *PISTILLATA*-like gene, *MADS12* (*Acc05042.1*). The ethylene treated fruit associated with the turquoise module contained three MICK MADS-like genes including the previously published *RIN/SEP4*-like gene (*MADS52 - Acc26640.1*) [15].

Other classes of well characterised TFs were examined, including the known floral determinacy genes described in a recent review [35]. By examining the closest kiwifruit homologues and module membership, it was possible to identify potential key genes such as the *APETALLA2*-like gene *AP2L11* (*Acc06022.1*), *CRABS CLAW* (*CRC*) *YAB3* (*Acc19364.1*) and *YAB4* (Acc06415.1), and *INNER NO OUTER* (*INO*) genes *YAB1* (*Acc08170.1* and *YAB2* (*Acc06179.1*). The three kiwifruit *NOZZLE* classes of *SPOREOCYTLESS* genes associated with ovule development were all located in the red module: *SPL1* (*Acc13721.1*), *SPL2* (*Acc19456.1*) and *SPL3* (*Acc21678.1*). Also the B3 class LAV genes had a clade expansion with *B3D47* (*Acc13067.1*), *B3D48* (*Acc30137.1*), *B3D49* (*Acc30138.1*), *B3D50* (*Acc30139.1*), *B3D51* (*Acc13066.1*), *B3D52* (*Acc06689.1*), *B3D53* (*Acc21264.1*), *B3D55* (*Acc31957.1*) and *B3D56* (*Acc11738.1*) all found in the red module. Additionally the *SHINY SHN* and *SHNL* ERF genes (*Acc12549.1* and *Acc17850.1*) associated with cuticular wax formation were identified. While these key genes were identified in the red module, it should be noted that, not all the best homologues to the genes identified in this review were present, indeed a large number were found in other coloured modules.

As the *AG* genes in other organisms have been shown to be the key carpel identity genes, the connectivity of the *AG* homologues *MADS28* and *MADS29* was examined further. Fifty-nine genes with a high (> 0.50 weight) association with the AG genes were identified and mapped (Fig. 6b). A network map of this subset shows a strong level of interdependency of these genes. Within this sub-network, genes that have been shown to be a direct target of *AG* in Arabidopsis were identified (Fig. 6b Orange). These include the aforementioned *SPL*, *CRC* and a *HEC2* like *bHLH* (*bHLH65*) genes.

Within the fruit ripening ethylene associated turquoise module there were a considerable number (29) of NAC TFs identified, including the previously described *NOR* like genes (*NAC1*, *NAC2*, *NAC3*) [36], as well as seven of the eight *EIN3-like* genes. This module also included 45 *ERF* genes [26], as well as *DOF4* [37, 38]. A previous studies of fruit ripening analysis [37] described 10 TFs associated with ripening (Additional data 1), most of which were located in other coloured modules suggesting that the wider study presented here gives a better resolution of tissue specific genes.

## Conclusion

In summary, we demonstrate that the eFP browser that we constructed and customised to display gene expression data, in combination with genome wide identification of the TFs and weighted gene co-expression network analysis provides a powerful platform for in-depth investigation of control and regulation of important processes in the plant life cycle and these tools can be easily customised to other fruiting species.

## Methods

### Plant material

All new tissue presented in this study (Additional data 3) were from the diploid *Actinidia chinensis* var. *chinensis* cv. ‘Hort16A’. These data were combined with previously published data from an *A. chinensis* var*. chinensis* cv. ‘Hort16A’ fruit maturation and ripening study [15], and a new comprehensive study of a bud series from the related tetraploid *Actinidia chinensis* var. *chinensis* cv. ‘Zesy002’ (Voogd et al., in preparation). Fruit was harvested from the Plant & Food Research site based at Kerikeri, New Zealand using standard pergola orchard management techniques. Tissues were harvested combining tissue from at least five different vines for each replicate. Between two and three replicates were taken for each tissue type, snap frozen in liquid nitrogen and stored at − 80 °C until needed.

### Gene mining, and comparisons

To identify a complete set of transcription factors a number of approaches were taken, for each approach, lists were generated and combined and filtered. In brief, a family was chosen to mine based on the classes identified in *Arabidopsis* [5]. The plant transcription factor database (http://planttfdb.cbi.pku.edu.cn/) which contains 2296 kiwifruit models generated from the original 2013 genome [18] were also assessed. Using an in house database (Bioview) platform, the manually annotated gene models [19] were identified containing the appropriate PFam domain [39]. Lastly, gene models identified from multiple BLASTP searches using appropriate diverse TFs matches were compared to the manually annotated gene sets. These were all aligned in geneious [40] using MUSCLE alignment [41]. Using the PFam domain to identify the DNA binding domain in the aligned regions, genes without a binding domain were removed, thus creating a complete list of TFs. For the R2R3 MYBs and WRKY family a few genes did not appear to have an Acc model. When this occurred, Web Apollo [42] was used to manually annotate an additional gene model and designated with either the Achn model number or a “no Acc match” in Additional data 1. The DNA binding domains were then aligned using PHYML [43], and named sequentially. Circle plots for Fig. 1 were generated in R using the “packcircles” library [44].

### RNA sequencing and transcriptomics

Total kiwifruit RNA was isolated using the Spectrum™ Plant Total RNA Kit (Sigma-Aldrich, St. Louis, MO, USA) and its integrity was assessed using the RNA 6000 Nano kit and the Agilent 2100 Bioanalyzer (Agilent Technologies, Santa Clara, CA, USA). Three RNA samples per sampling time were used for subsequent library construction and sequencing. The sequencing libraries were constructed according to the TruSeq RNA sample preparation guide (Illumina, San Diego, CA, USA) and subsequently sequenced by a HiSeq 2500 Sequencing System (Illumina), obtaining paired-reads of 125 bp. Library construction and sequencing were performed at Macrogen (www.macrogen.com). A minimum of 16 M reads were obtained from the Hort16A tissues and percentage read mapping ranging from 69 to 92% Additional data 3. The Zys002 bud series had a minimum of 11.9 M reads and percentage read mapping was 51–79%. All raw sequence data can be found in the NCBI RNA-seq depositories detailed in Additional data 3. The raw reads were aligned using Spliced Transcripts Alignment to a Reference (STAR) (version 2.5.2a) on default settings [45] to the *A. chinensis* Red 5 v1.69 gene models [19]. PCA analysis was undertaken with the plotPCA in the DeSeq2 package in R [46] using the 5000 most differentially expressed genes (ntop = 5000). Raw reads were normalised to transcripts per million (TPM) (Additional data 4).

### Development of eFP browser

The eFP browser [1] was generated using deposited code from SourceForge (https://sourceforge.net/projects/efpbrowser/). Template images were generated by the graphic design team at Plant & Food Research. The template images were colourised and reformatted for the eFP browser using the GIMP (https://www.gimp.org/) version 2.8.22 software program. Several additional features were added to the eFP source code. The modified source code is available via GitHub (https://github.com/pfrnz/eFP-Browser). Experiment images are constructed from component images described by the XML file for each section. Multiple sections can be included in each experiment view. This allows the reuse of component images for different sections and experiment views, greatly reducing the effort required to add new experiment views. Auto-completion of gene ID input on the interface was implemented with JavaScript and PHP, referencing the existing ID look-up SQL table. The eFP browser was containerised using Docker. The Dockerfile builds a Docker container which incorporates the software requirements, and eFP Browser source code and experiment images. A second Docker container was used for the SQL database of read numbers expression data. A Docker-Compose file is included to run the eFP Browser and SQL containers. The Actinidia eFP browser was set up on the Bio-Analytic Resource for Plant Biology server at bar.utoronto.ca and is available at http://bar.utoronto.ca/efp_actinidia/cgi-bin/efpWeb.cgi

### WGCNA network analysis

RNA-seq data for gene models associated with transcription factors were extracted and transcription factors that were not expressed (based on no read alignments) in any of the samples were removed from the analysis. Weighted gene co-expression network analysis (WGCNA) [30] v 1.68 was undertaken in the R environment v3.5.1. In the WGCNA environment the soft power was calculated and set to six. The minimum module size was set to 30 and the merge cut height set to 0.25. Hub genes for each colour environment were calculated using “chooseTopHubInEachModule”. Network data were exported into Cytoscape v3.7.1 for visualisation.

## Supplementary Information

**Additional file 1: Additional data 1**. Table of transcription factors, with corresponding colour module assignments and connectivity.

**Additional file 2: Additional data 2**. Phylogenetic alignments of DNA binding sites for Transcription factor classes with over nine genes.

**Additional file 3: Additional data 3**. Details of the RNA-seq data used in this study.

**Additional file 4: Additional data 4**. Table of TPM for the RNA-seq data.

**Additional file 5 : Additional data 5**. Analysis of circadian gene expression.

**Additional file 6: Additional data 6**. Numbers of transcription factor classes within each colour module.

## Data Availability

All RNA-seq data can be found in NCBI bioproject PRJNA691387 study SRP301347, accession numbers SRR13413552: SRR13413581 (details in Additional Data 3). The Actinidia eFP browser can be found at http://bar.utoronto.ca/efp_actinidia/cgi-bin/efpWeb.cgi

## Abbreviations

eFP: Electronic Fluorescent Pictograph
TF: Transcription factor
*WGCNA*: Weighted gene co-expression network analysis
