# Supplementary material for: A gene expression atlas for kiwifruit (Actinidia chinensis) and network analysis of transcription factors
Source: BMC Plant Biol. 2021 Feb 27;21:121. doi: 10.1186/s12870-021-02894-x (PMC7913447; doi:10.1186/s12870-021-02894-x)
Supplement: Supplementary file 2 — Additional file 2: Additional data 2. Phylogenetic alignments of DNA binding sites for Transcription factor classes with over nine genes. [file 12870_2021_2894_MOESM2_ESM.docx]

Contents

[Zn fingers 3](#_Toc30512867)

[C2H2 3](#_Toc30512868)

[CO/DBB 4](#_Toc30512869)

[DOF 4](#_Toc30512870)

[C3H 4](#_Toc30512871)

[SQBP 6](#_Toc30512872)

[GATA 6](#_Toc30512873)

[VOZ 6](#_Toc30512874)

[LSD 6](#_Toc30512875)

[NF 6](#_Toc30512876)

[SRS 7](#_Toc30512877)

[MYB domains 8](#_Toc30512878)

[MYB related 8](#_Toc30512879)

[R2R3 9](#_Toc30512880)

[R2R3 continued 10](#_Toc30512881)

[ARR 11](#_Toc30512882)

[G2/GARP 11](#_Toc30512883)

[BHBH 11](#_Toc30512884)

[BHLH 11](#_Toc30512885)

[TCP 11](#_Toc30512886)

[AP2 domain 11](#_Toc30512887)

[ERF 11](#_Toc30512888)

[AP2 11](#_Toc30512889)

[HB domain 12](#_Toc30512890)

[HD-ZIP 12](#_Toc30512891)

[WOX 12](#_Toc30512892)

[HDPHD 12](#_Toc30512893)

[TALE 12](#_Toc30512894)

[HB only 12](#_Toc30512895)

[ZF-HD 12](#_Toc30512896)

[B3 domain 12](#_Toc30512897)

[ARF 12](#_Toc30512898)

[RAV 12](#_Toc30512899)

[LAV/B3 12](#_Toc30512900)

[BZIP domain 12](#_Toc30512901)

[BZIP 12](#_Toc30512902)

[MADS domain 12](#_Toc30512903)

[TYPE1 MADS 12](#_Toc30512904)

[MICK - MADS 12](#_Toc30512905)

[HMG type 12](#_Toc30512906)

[HMG 12](#_Toc30512907)

[ARID 12](#_Toc30512908)

[YABBY 12](#_Toc30512909)

[NAC 13](#_Toc30512910)

[WRKY 14](#_Toc30512911)

[TRIHELIX 14](#_Toc30512912)

[FAR 14](#_Toc30512913)

[GRAS 14](#_Toc30512914)

[BCP 14](#_Toc30512915)

[BES 14](#_Toc30512916)

[LFY 14](#_Toc30512917)

[EIL 14](#_Toc30512918)

[CATMA 14](#_Toc30512919)

[EF2 14](#_Toc30512920)

[CPP 14](#_Toc30512921)

[GRF 14](#_Toc30512922)

[GeBP 14](#_Toc30512923)

[HST 14](#_Toc30512924)

[LBD 14](#_Toc30512925)

[NZZ 14](#_Toc30512926)

[NIN 14](#_Toc30512927)

[S1Fa 14](#_Toc30512928)

[SAP 15](#_Toc30512929)

[HRT 15](#_Toc30512930)

[WHIRLY 15](#_Toc30512931)

## Zn finger domains

### C2H2

### CO/DBB

### DOF

###

### C3H

### SQBP

### GATA

### VOZ

4 genes

### LSD

9 genes

### NF

NFYa

NFYb

### SRS

## MYB domains

### MYB related and GARP


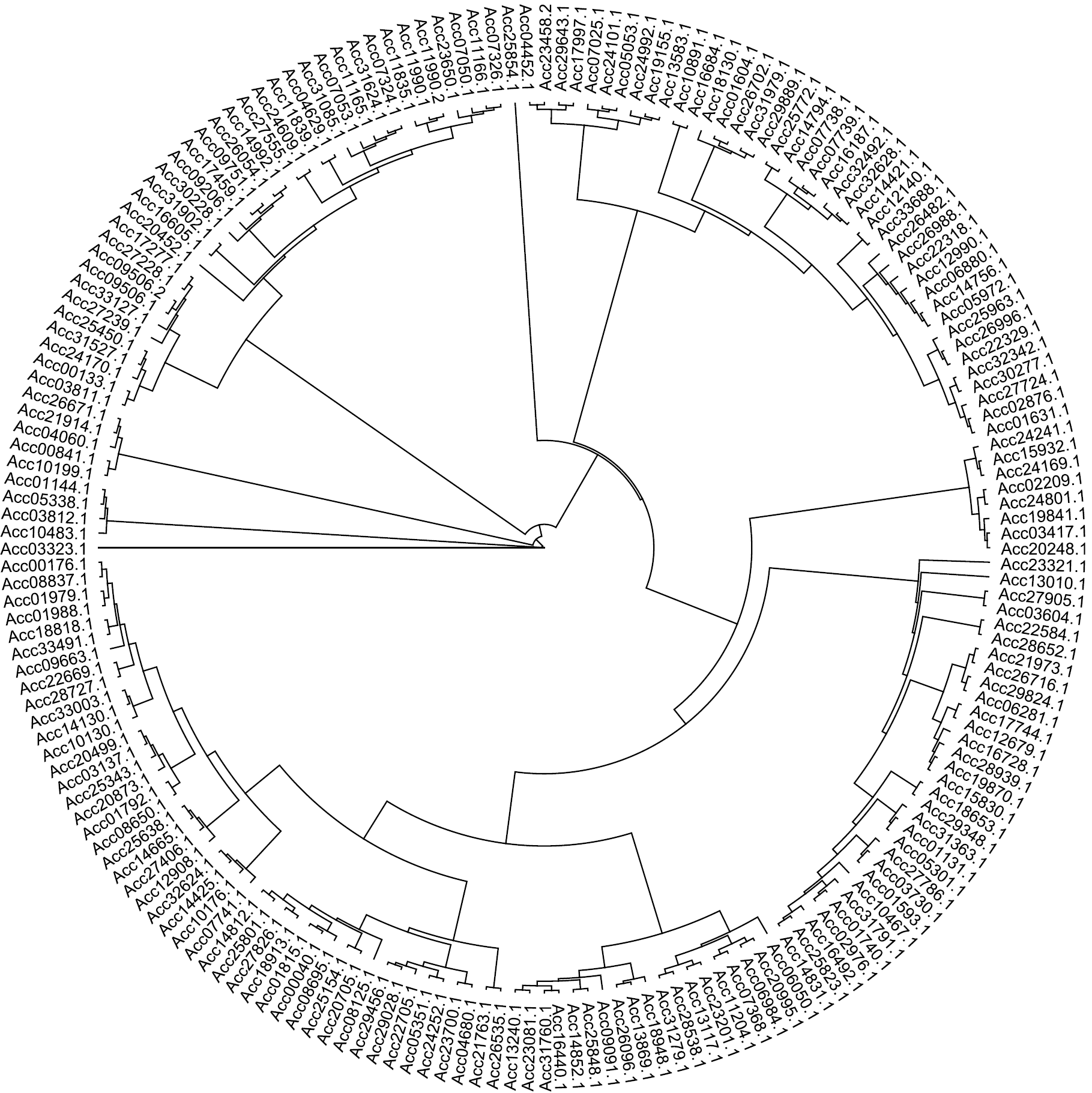


### R2R3 MYB

### ARR

##

## BHLH domain

### BHLH

### TCP

## AP2 domain

See supplemental data in Gunaseelan K, McAtee PA, Nardozza S, Pidakala P, Wang R, David K, et al. (2019) Copy number variants in kiwifruit ETHYLENE RESPONSE FACTOR/APETALA2 (ERF/AP2)-like genes show divergence in fruit ripening associated cold and ethylene responses in C-REPEAT/DRE BINDING FACTOR-like genes. PLoS ONE 14(5):e0216120. <https://doi.org/10.1371/journal.pone.0216120>

## HB domain

###

## B3 domain

### ARF – kiwifruit (green) aligned to Arabidopsis (Black) and Tomato (red) ARF proteins


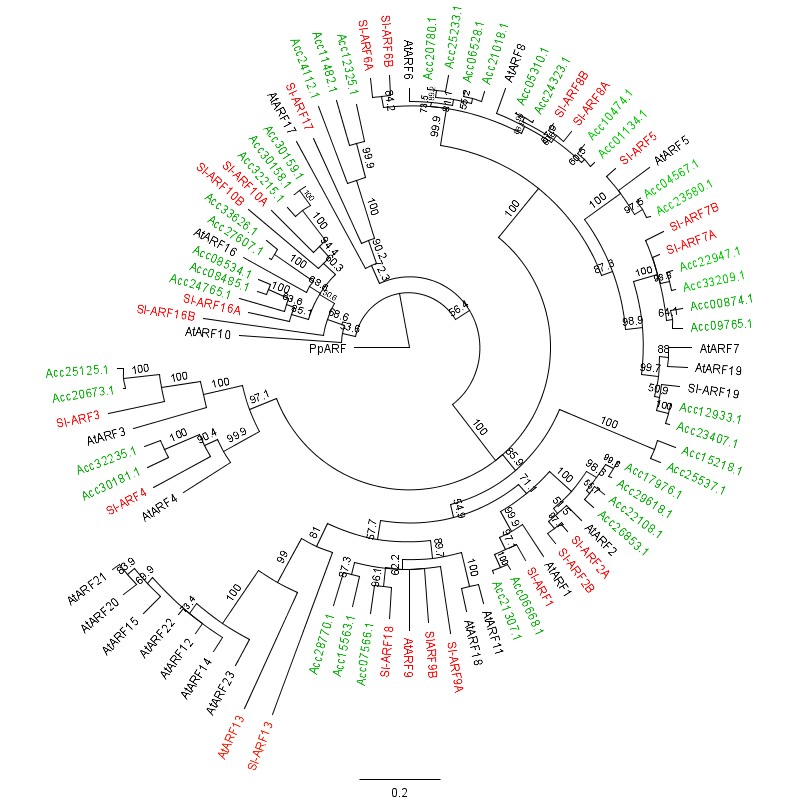


###

### RAV

###

### LAV/B3

## BZIP domain

### BZIP

##

## MADS domain

See figure 2 in paper

## HMG type

### HMG, ARID and YABBY

## NAC

## WRKY

## TRIHELIX

## FAR

1 gene

### GRAS

##

## BCP

##
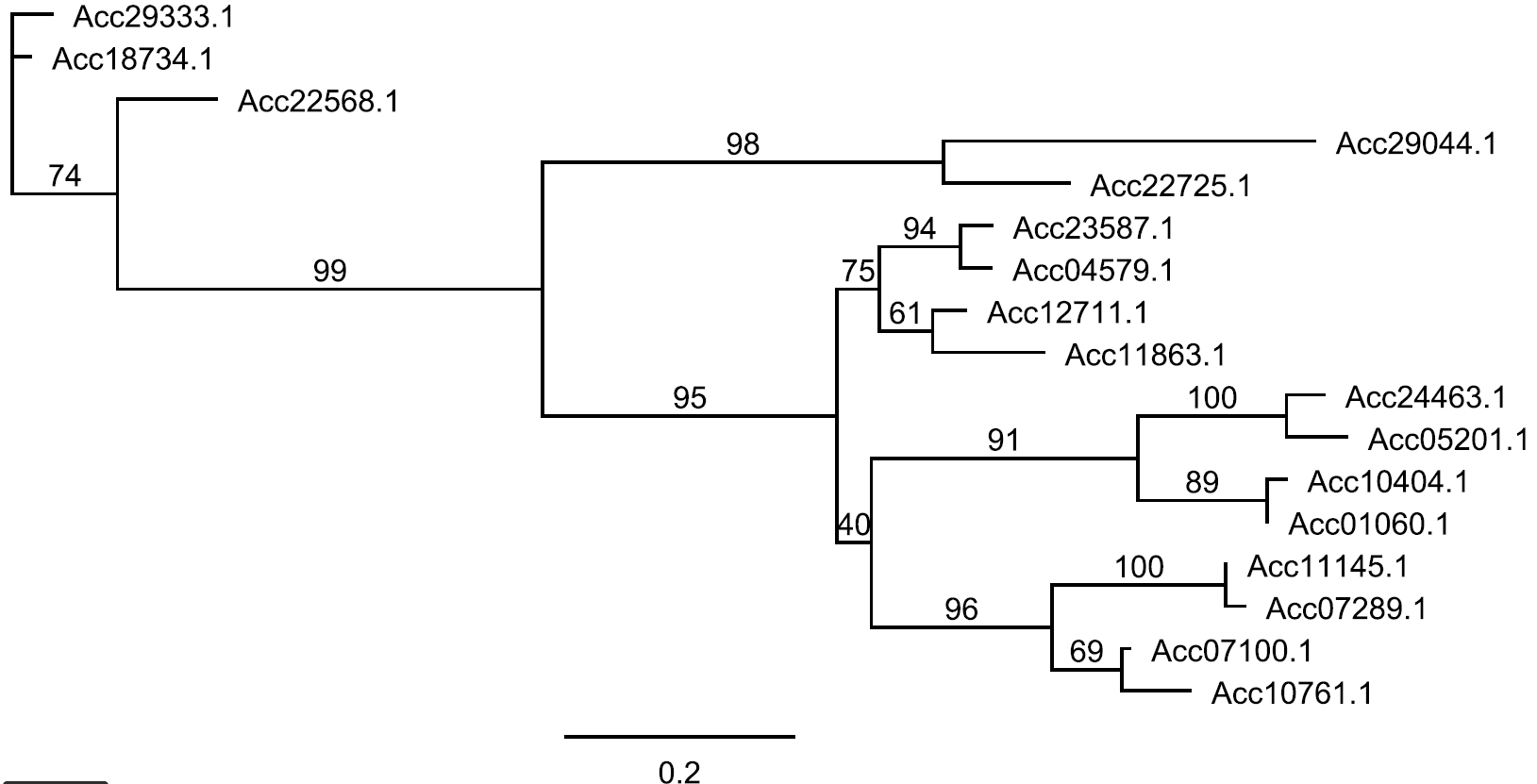


## BES

##
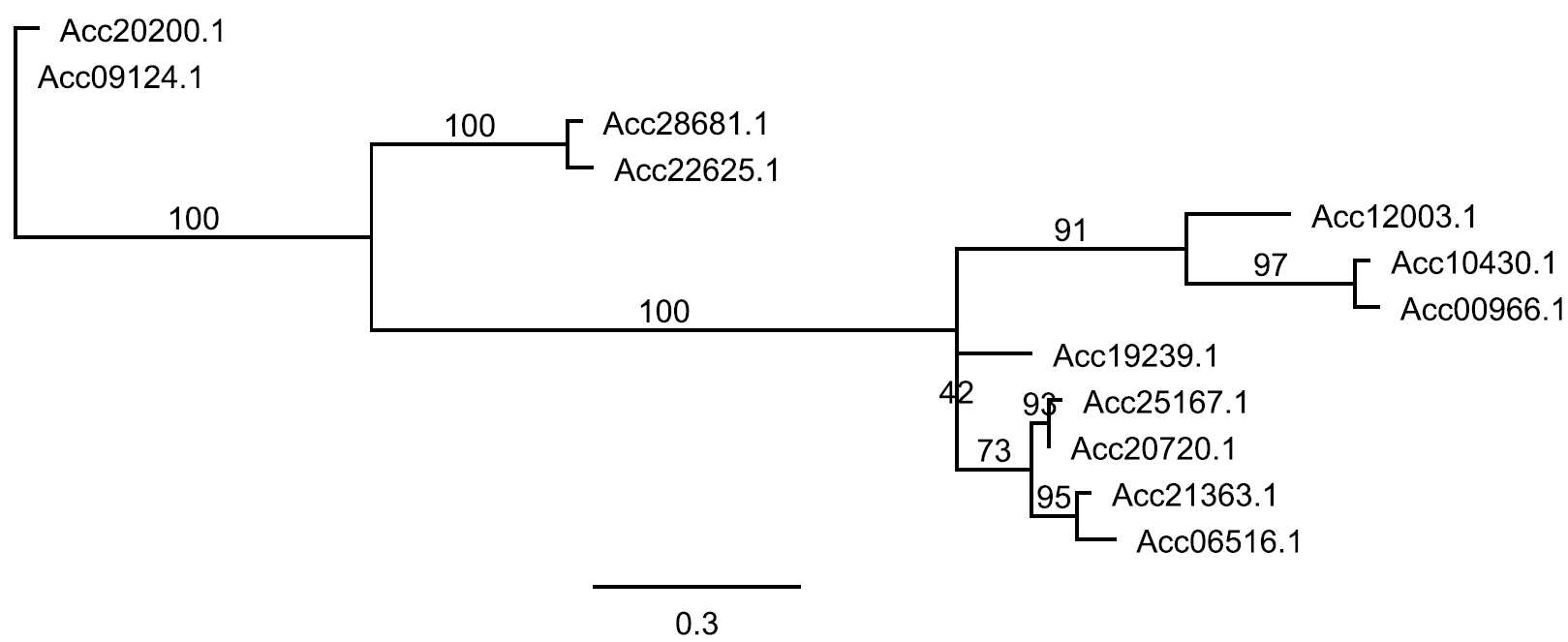


## LFY

2 genes

## EIL

## CATMA

## EF2

## CPP

## GRF

## GeBP

7 genes

## HST

## LBD

## NZZ

3 genes

## NIN

## S1Fa

3 genes

## SAP

2 genes

## HRT

1 gene

## WHIRLY

4 genes

## PLATZ
