# Supplementary material for: A gene expression atlas for kiwifruit (Actinidia chinensis) and network analysis of transcription factors
Source: BMC Plant Biol. 2021 Feb 27;21:121. doi: 10.1186/s12870-021-02894-x (PMC7913447; doi:10.1186/s12870-021-02894-x)
Supplement: Supplementary file 5 — Additional file 5 : Additional data 5. Analysis of circadian gene expression. [file 12870_2021_2894_MOESM5_ESM.docx]

Additional data 5

Two MYB related genes most closely similar to the *LATE ELONGATED HYPOCOTYL* (*LHY*) gene. Both show a high similarity of expression. MYBR92 has the highest expression and therefore selected as representing *LHY* like gene.

Two GIGANTEA like genes, with a similar expression pattern. The higher expressed Acc12229.1 was selected as a representative GI like gene.
